# Study on Boiling Heat Transfer Characteristics of Composite Porous Structure Fabricated by Selective Laser Melting

**DOI:** 10.3390/ma16196391

**Published:** 2023-09-25

**Authors:** Houli Liu, Zhonghao Gu, Jun Liang

**Affiliations:** 1 School of Mechanical and Power Engineering, East China University of Science and Technology, Shanghai 200237, China; 2 College of Chemistry and Materials Science, Anhui Normal University, Wuhu 241002, China

**Keywords:** selective laser melting, composite porous structure, heat transfer, pool boiling

## Abstract

Surface porosity is an important means of enhancing boiling heat transfer. In this paper, two kinds of composite porous structures of surface micropore + square channel and framework micropore + square channel were prepared by selective laser melting technology using AlSi10Mg as the powder material. The effect of composites with different pore forms on boiling heat transfer was investigated in pool boiling experiments. It was found that controlling the thickness of the powder layer manufactured by selective laser melting can change the surface roughness of the sample, and the sandblasting treatment reduced the surface roughness of the samples. The average heat transfer coefficient of the rough surface composite porous structure sample was increased by 40% compared to the sandblasted sample. The micropores on the surface of the sample and inside the framework significantly enhanced the heat transfer coefficient of the composite porous structure. The presence of surface micropores increased the heat transfer area and the vaporization core density of the composite porous structure and exhibited excellent heat transfer coefficient improvement in the low heat flux region. The framework microporous composite porous structure can form effective gas–liquid diversion at high heat flux and obtain higher heat transfer performance. The large channel in the composite porous structure is the key control factor of the critical heat flux.

## 1. Introduction

With the continuous upgrading of electronic chips towards miniaturization and precision, performance improvement also puts forward higher demands for heat dissipation. Traditional single-phase convection is no longer sufficient to meet the heat dissipation requirements of high-power electronic equipment, and boiling heat transfer can be used to effectively control the heat generation of electronic equipment using its phase change characteristics, which have a broader application prospect [1].

In general, three types of enhanced structural surfaces are used in enhanced boiling applications: “pore and channel” surfaces, reentrant cavities and fin surfaces, and porous coating surfaces. The preparation methods for these porous structures primarily involve machining (wire cutting, CNC machining, MEMS, etc.) [2,3,4,5], sintering [6,7,8], electrochemical methods (vapor deposition, electrochemical deposition, anodic oxidation, etc.) [9,10,11], laser processing [12,13], etc. All of the above methods are devoted to improving the heat transfer coefficients (HTC) and critical heat flux (CHF). Changes in the surface state can significantly affect the specific surface area, wettability, and vaporization core density of the boiling heating surface; thus, these are crucial for enhancing boiling heat transfer. However, these surface porous methods can only improve one aspect of heat transfer and fail to achieve a comprehensive improvement. Composite porous structures can combine the advantages of different scales of pores to achieve an overall improvement in heat transfer.

More studies have been carried out on enhanced boiling heat transfer based on composite porous structures. The sintering porous method is one of the main methods for fabricating composite porous structures. Cora et al. [14] prepared modulated microporous surfaces by sintering and studied the parameters, such as different powder particle sizes and sintering temperatures. The porosity of the modulated porous layer they manufactured ranges from 15% to 40%, and pool boiling experiments have confirmed the potential of the modulated porous layer to enhance heat transfer. Ha and Graham [15] have processed different forms of microchannels based on sintered microlayers, hoping to reduce the escape resistance of bubbles through microchannels. In their study, CHF and HTC were found to increase with increasing microchannel depth and decreasing channel spacing. The preparation of microporous layers on top of machined channels by deposition or sintering is another manufacturing method to form composite porous structures. Fully utilizing the advantages of micro/nano pores and macro-porous channel structures to enhance boiling heat transfer, this method has always been favored by researchers. Kandlikar [16,17] sintered microporous layers at different parts of machined channel structures to construct composite porous structures. The initial nucleation provided by the microporous layer and the gas–liquid separation of the channel structure were utilized. They found that the composite porous structure can obtain a relatively high heat flux at a lower wall superheat. Chen et al. [18] were inspired by the leaf vein bionic structure to fabricate the channel structure and prepared nanowires on top of the structure for enhanced boiling heat transfer. The results show that the macro-porous channel structure is beneficial to reduce the gas–liquid flow resistance, and the nanowire structure increases the nucleation site; a maximum CHF increase of 206% was obtained. Shakeri et al. [19] fabricated bi-conductive surfaces by electrodeposition and verified the heat transfer properties of this composite porous structure by pool boiling experiments. Research has found that the optimal bi-conductive surfaces increase by 62% and 260%, respectively, compared to plain CHF and HTC. Der et al. [20] constructed a serpentine channel structure based on the design parameters of the hydraulic diameter and verified the influence of structural parameters on phase change heat transfer.

Although the composite porous structure can comprehensively improve the heat transfer effect, the process of preparing such structures is often relatively complex, requiring collaboration with molds and various processing methods for successful manufacturing. The uncontrollable pore structure limits the further improvement of heat transfer enhancement.

Selective laser melting (SLM) technology is a new type of metal additive manufacturing method, which has significant advantages in the controllable preparation of complex configurations. SLM technology can form micropores on the surface of the machined parts or inside the skeleton due to its laser processing characteristics. The existence of the micropore layer significantly enhances heat transfer. In recent years, some scholars have applied selective laser melting forming technology to the field of enhanced heat transfer [21,22,23,24]. Wong et al. [25] designed lattice samples with different structural dimensions for the experimental study of pool boiling heat transfer, and the HTC obtained was 2.81 times higher than that of the smooth surface. Liu et al. [26] used SLM technology to prepare composite porous structures with different thicknesses to enhance boiling heat transfer. Their research found that the CHF of composite pore samples with a height of 1.5 mm reached 3.2 times that of smooth surfaces. Zhang et al. [27] investigated the effect of grid structures with different pore sizes on pool boiling heat transfer characteristics and found that as the pore size increased, the sample surface area decreased, leading to a reduction in the heat transfer coefficient.

The above literature analysis shows that SLM molding can prepare composite porous structures with different pore forms by controlling the printing parameters in the SLM manufacturing process due to its fabrication characteristics. However, the existing literature on the mechanism of enhancing boiling heat transfer by manufacturing different forms of composite porous structures in SLM has not yet been addressed. Therefore, this study aims to fill this gap by designing and manufacturing composite porous structure specimens with different pore forms by controlling the printing process parameters of SLM. The pore structure characteristics of the sample were analyzed, and the effect of different pore structures on boiling heat transfer characteristics was investigated using the pool boiling method.

## 2. Materials and Methods

### 2.1. Design and Manufacture of Samples

The SLM technology used in this paper to fabricate porous specimens is described in the literature [28], and the metal powders were made from AlSi10Mg. The pore structure of the prepared sample is a composite porous structure with two pore forms: micropores on the surface of the solid framework and micropores inside the framework. The sample with micropores on the surface of the solid framework is named “SS” based on the sample + surface. Samples with micropores inside the framework are named “SF” according to the sample + framework. We controlled the 3D printing preparation process parameters for the fabrication of different forms of pore structures. The sample adopts a grid pore structure design, and the square pore structure design refers to the design method of Zhang [27]. Each square pore measures 1.1 mm, with a sample wall thickness of 0.5 mm and a consistent height of 1.5 mm across all samples, and the overall size of the sample is 12 mm × 12 mm. The unified sample structure size was prepared, and the forming quality of the square pore structure was changed by controlling the parameters, such as printing power, printing speed, and printing layer thickness, to investigate the effect of different microporous structures on boiling heat transfer. The printing process parameters of the SS-1 and SS-2 samples are the same. However, the SS-2 samples were sandblasted to compare the effects of different surface characteristics on boiling heat transfer. The selection of the printing process parameters in this article can be found in the literature [26]. A total of 4 sets of samples with different parameters were designed, and the specific printing parameters of the samples are shown in Table 1.

### 2.2. Experimental Apparatus and Data Reduction

**Figure 1:**
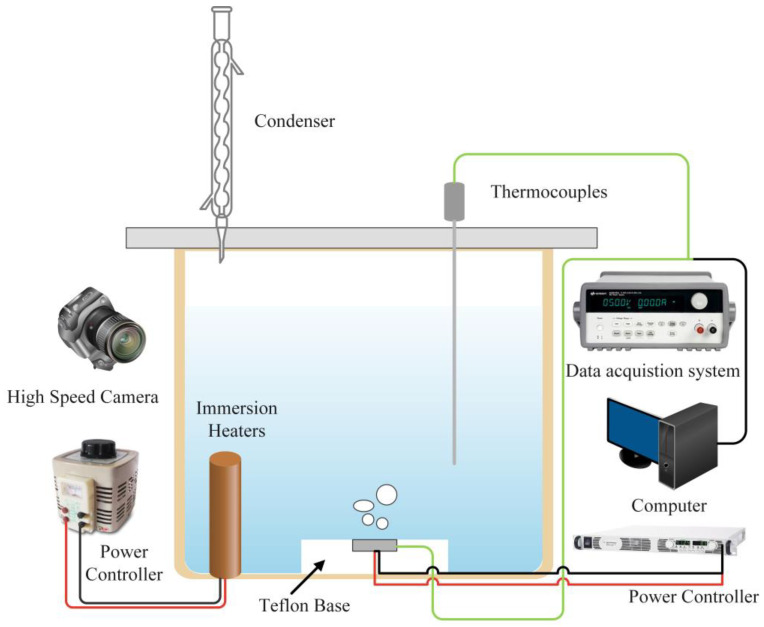
Schematic of pool boiling facility.

The schematic diagram of the pool boiling experimental device is shown in Figure 1. The dimensions of the boiling pool chamber are 150 mm × 150 mm × 200 mm, and it is composed of organic glass. A ceramic heating plate embedded on a PTFE substrate is installed on the bottom surface of the cavity, with dimensions of 12 mm × 12 mm × 2.5 mm. The ceramic heating plate is produced by Watlow (ULTRAMIC® WALN-6, from Watlow^®^, St. Louis, MO, USA), and its roughness is less than 1.5 μm. The input heating power is provided by Agilent N5769A (Palo Alto, Santa Clara, CA, USA) programmable DC power supply. A K-type thermocouple is embedded inside the heating plate to detect the temperature of the heating surface.

Due to the inconvenience of welding ceramic and aluminum alloy samples, the experimental samples were fixed on the heating surface by a thin steel wire similar to the method employed by Aznam [29]. Condensers and auxiliary heating rods were arranged at the top and bottom of the boiling pool, respectively, which were used to condense the water vapor generated by the experiment and heat the deionized water to make it boil. Before each experiment, we turned on the auxiliary heating rod and heated the deionized water to boiling for 1 h to remove non-condensable gas from the water. In addition, a T-type thermocouple was installed near the sample to detect changes in the water temperature. The movement of bubbles in the experiment was recorded using the high-speed camera (MotionXtra N4, from IDT Inc., San Jose, CA, USA).

**Figure 2:**
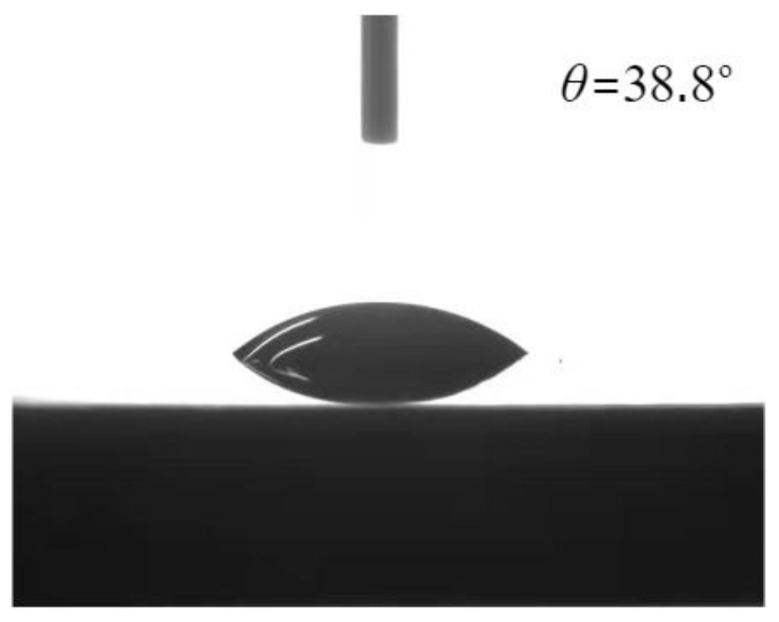
Smooth surface contact angle measurement with deionized water.

For the purpose of characterizing the surface wettability, 5 μL of deionized water was used to measure the static contact angle at room temperature. Video capture and contact angle analysis were performed by goniometer (Kruss, DSA30, Hamburg, Germany). The static contact angle of the smooth surface was 38.8°. The contact angle is illustrated in Figure 2.

The heat flux $q^{\prime \prime }$ in the experiment is calculated using Equation (1).

(1) $$q^{\prime \prime }=\frac{U\times I}{A}$$

where *U* and *I* are the voltage and current of the heating power supply, respectively, and A is the heating area.

The heat transfer coefficient h is calculated by Equation (2).

(2) $$h=\frac{q^{\prime \prime }}{T_{w}-T_{sat}}$$

where *T_w_* is the boiling surface temperature and *T_sat_* is the saturation temperature at 1 atm.

(3) $${\left(\Delta q^{\prime \prime }\right)}^{2}={\left(\frac{\partial q^{\prime \prime }}{\partial U}\right)}^{2}{\left(\Delta U\right)}^{2}+{\left(\frac{\partial q^{\prime \prime }}{\partial I}\right)}^{2}{\left(\Delta I\right)}^{2}+{\left(\frac{\partial q^{\prime \prime }}{\partial A}\right)}^{2}{\left(\Delta A\right)}^{2}$$

(4) $$\frac{\Delta q^{\prime \prime }}{q^{\prime \prime }}=\sqrt{{\left(\frac{\Delta U}{U}\right)}^{2}+{\left(\frac{\Delta I}{I}\right)}^{2}+{\left(\frac{\Delta A}{A}\right)}^{2}}$$

(5) $${\left(\Delta h\right)}^{2}={\left(\frac{\partial h}{\partial q^{\prime \prime }}\right)}^{2}{\left(\Delta q^{\prime \prime }\right)}^{2}+{\left(\frac{\partial h}{\partial T_{w}}\right)}^{2}{\left(\Delta T_{w}\right)}^{2}+{\left(\frac{\partial h}{\partial T_{sat}}\right)}^{2}{\left(\Delta T_{sat}\right)}^{2}$$

(6) $$\frac{\Delta h}{h}=\sqrt{{\left(\frac{\Delta q^{\prime \prime }}{q^{\prime \prime }}\right)}^{2}+{\left(\frac{\Delta T_{sat}}{T_{w}-T_{sat}}\right)}^{2}+{\left(\frac{\Delta T_{w}}{T_{w}-T_{sat}}\right)}^{2}}$$

The uncertainty analysis was performed using the calculation method proposed by Kline [30]. There are two types of errors: one for precision and the other for bias. Precision errors arise from the sensitivity or fluctuations, and bias errors arise from the resolution or calibration. In the present study, the measurement uncertainties for *U* and *I* are 0.012% and 0.2%, respectively. The uncertainty of the thermocouple temperature is less than 0.5 K. According to the calculation of uncertainty caused by the voltage, current, and thermocouple detection, the maximum uncertainty of the heat flux is 5%, and the maximum uncertainty of the heat transfer coefficient is 8%.

## 3. Results

### 3.1. Analysis of Pore Characteristics of Samples

**Figure 3:**
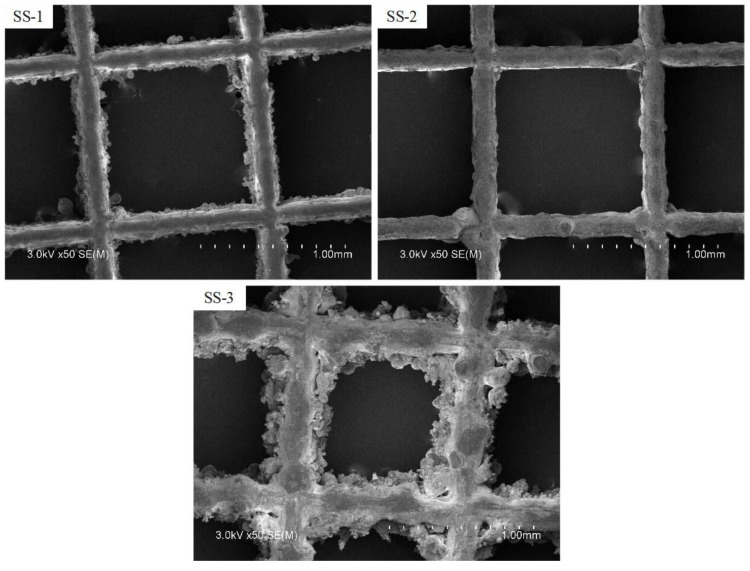
SEM of composite porous samples with different surface micropores.

The surface morphology of surface microporous composite porous samples, prepared under different printing process conditions, was analyzed. Sample SS-1 and sample SS-2 were designed using the same printing process (Table 1), where the SS-2 sample was sandblasted to remove bonded, unmelted metal powder from the surface. The SEM photos of the three groups of samples are shown in Figure 3. The SS-1 sample has better forming quality, and only a small amount of unmelted powder is bonded on the surface. The SS-2 sample is the surface morphology of the sample after sandblasting. The powder on the surface after sandblasting is removed, and the surface is relatively smooth.

It can be seen from Figure 3 that the surface of the SS-3 sample is comparatively rougher because the thickness of the printing powder layer of this sample is twice that of the SS-1 sample. Under the condition that other parameters remain unchanged, the increase in the layer thickness leads to the increase of unmelted powder adhesion on the surface to form a good microporous layer, and the relatively rough surface is often beneficial to boiling heat transfer. Based on the above 3D-printed sample morphology analysis, it can be seen that increasing the thickness of the powder layer can increase the powder bonding on the surface of the porous sample, thereby forming a layer of sintered microporous layer on the surface of the sample skeleton.

**Figure 4:**
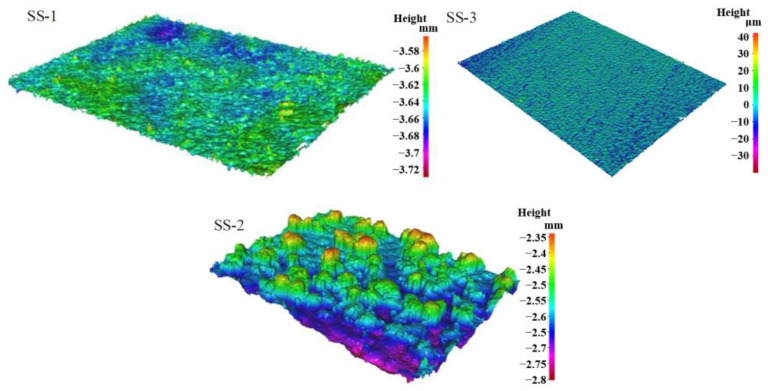
Surface morphology analysis of different samples.

The surface characteristics of 3D-printed samples were analyzed using a three-dimensional profilometer (InfiniteFocus G4), as depicted in Figure 4. The sample is placed in the lens aperture range of the three-dimensional profilometer stage, and the sampling software is set up for surface scanning. The three-dimensional profilometer will automatically zoom to complete the sampling of the surface morphology according to the surface characteristics of the sample. It can be seen that the surface morphology of the sample is affected due to process limitations, and the surface of the sample skeleton is uneven, which corresponds to the SEM photographs in the previous section. Because of the sandblasting treatment, the surface of the SS-2 sample is smoother than that of the other two groups, while the surface morphology of the SS-1 sample without sandblasting treatment shows obvious roughness. The surface of sample SS-3 shows obvious mountain-like protrusions and the surface is rougher.

This phenomenon largely originates from semi-melted powder particles that adhere to the sample’s surface during the printing process. These semi-melted powders are bonded together to form a microporous layer on the surface of the printed sample.

To elucidate the surface roughness characteristics of samples fabricated under varying process conditions, a three-dimensional profiler was employed for measuring the surface roughness. The rough surface is beneficial to the enhancement of boiling heat transfer. The measured roughness values for the SS-1, SS-2, and SS-3 samples were 12.1 μm, 5.6 μm, and 23.7 μm, respectively. Notably, the SS-3 sample exhibited the highest surface roughness among all samples, which is consistent with the findings from the three-dimensional morphology analysis presented in Figure 4. The thickness of the powder layer has an important influence on the surface roughness of the sample.

**Figure 5:**
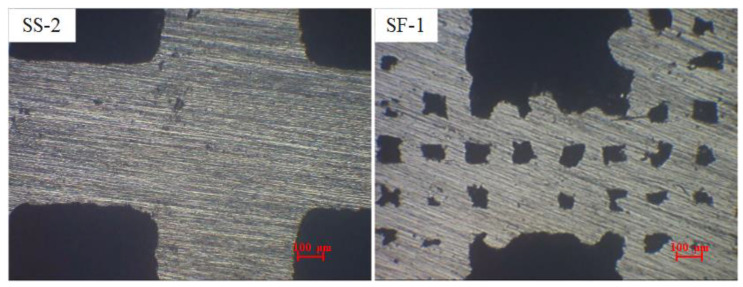
Pore characteristics in composite porous framework.

In addition to scrutinizing the surface morphology and roughness characteristics of the printed sample, Figure 5 shows the metallographic photos of the SS-2 and SF-1 samples to facilitate the analysis of the internal pore characteristics of the porous sample skeleton. The SS-1 sample in Figure 5 was prepared using a small scanning spacing process. It can be seen that the composite pore skeleton is dense and that it does not contain micron pores. In contrast, the SF-1 sample was prepared using a large scanning spacing method, and the sample skeleton contained micropores. By controlling the scanning distance of 3D printing and combining it with the design of macro-sized pores, the controllable preparation of composite porous structures featuring micron-sized pores and millimeter-sized pores is achieved.

### 3.2. Comparison of Heat Transfer between Surface Microporous Composite Porous Samples

**Figure 6:**
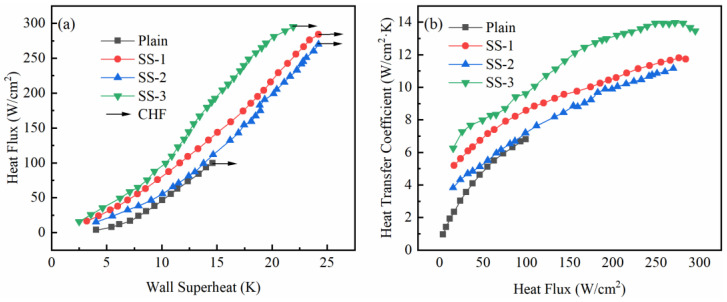
Heat transfer comparison of composite porous samples with different surface micropore characteristics: (**a**) heat flux curves; (**b**) heat transfer coefficient curves.

To compare the difference in pool boiling heat transfer of surface composite porous structures under different printing process parameters, the heat flux curves of the surface microporous composite porous structures are given in Figure 6a. It is evident from Figure 6a that the SS-3 sample demonstrated the best heat transfer enhancement effect. The sample consistently achieved a higher heat flux for the same wall superheat. The heat flux curve of SS-1 was located on the right side of SS-3, indicating that its heat transfer enhancement ability was weaker than that of SS-3. The heat transfer performance of SS-2 was consistently worse than that of the other two groups of samples throughout the experiment, which was determined by its surface characteristics. Although the sample was prepared by the same process as the SS-1, the surface became smoother after the sandblasting treatment. The vaporization core formed by the microporous layer was removed so that the heat flux curve was always on the right side of the other two groups of samples. Comprehensively, it becomes apparent that although discernible differences existed in the overall heat flux curves among the three sample groups during the experiment, these dissimilarities did not manifest prominently in the final CHF values. This shows that under the premise of the same square pore size, the difference in the surface roughness is not the key to determining the final CHF.

The HTC curve of the composite porous sample with a microporous layer on the surface is shown in Figure 6b. It can be seen from Figure 6b that the HTC of the surface microporous composite porous structure samples are all larger than the smooth surface, indicating that adding porous structures above the smooth surface can enhance its boiling HTC. Notably, the HTC of the SS-3 sample is higher than that of the other two samples, which is more obvious in the high heat flux region. Due to the removal of the surface microporous layer after sandblasting, the HTC of the SS-2 sample is always lower than that of SS-1. By adjusting the printing process parameters, the reliable formation of the microporous layer on the surface of the composite porous structure sample can be achieved. The presence of the surface microporous layer enhances the HTC of the composite porous sample.

### 3.3. Comparison of Heat Transfer between Composite Porous Structures with Different Pore Forms

**Figure 7:**
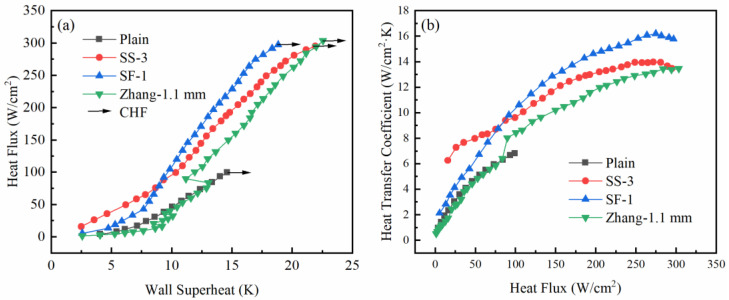
Comparison of heat transfer of composite porous samples with different pore forms: (**a**) heat flux curves; (**b**) heat transfer coefficient curves.

The influence of different surface characteristics of the composite porous structure on the boiling heat transfer performance is analyzed in the previous section. In this section, the heat transfer results of the composite porous structure with two different pore forms are compared. Figure 7a illustrates the comparison of the heat flux curves of SS-3 and SF-1, and the experimental results of Zhang’s [27] grid pore sample are also compared.

It can be seen from Figure 7a that the heat flux curves of the three samples are significantly different. In the low heat flux region, the heat flux of SS-3 was greater than that of SF-1 under the same wall superheat, and the surface microporous composite porous junction had better heat transfer ability. Under high heat flux conditions, the heat flux curve of SF-1 was located on the left side of SS-3, indicating that the framework microporous composite porous structure can achieve better heat transfer performance under high heat flux conditions. The grid pore size used by Zhang [27] was also 1.1 mm, but its heat flux curve was located on the right side of the current research throughout the entire heat transfer process, and its heat transfer performance was lower than that of the current composite porous structure. The CHF of the composite porous structure with two different pore forms was not significantly different, and its value was slightly lower than Zhang’s result.

Figure 7b shows the comparison of HTC curves of composite porous samples with different pore forms. The current HTC of the composite porous samples was higher than Zhang’s experimental results. In the low heat flux area, the HTC of the composite porous sample with micropores on the surface was higher. However, under the high heat flux region, the HTC was lower than that of the composite porous sample with micropores in the skeleton. The reasons for the difference in heat transfer between the two pore forms of composite porous structures will be analyzed later.

### 3.4. Analysis of Heat Transfer Enhancement Mechanism with Different Pore Structures

**Figure 8:**
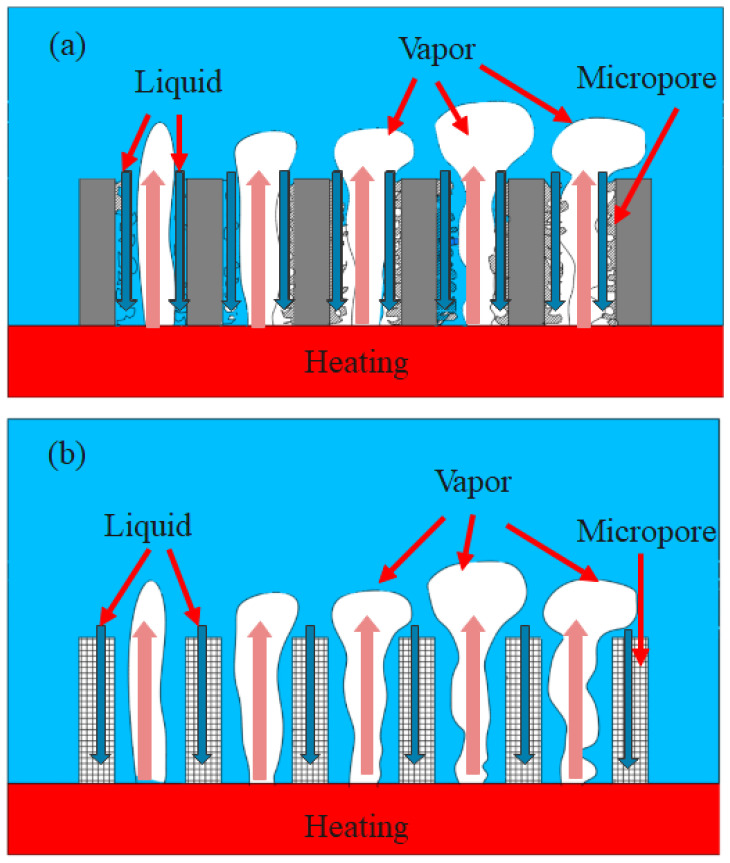
Schematic diagram of gas–liquid flow in different forms of composite porous structure. (**a**) Surface microporous composite porous structure. (**b**) Framework microporous composite porous structure.

The pore structure of the composite porous structure is the main reason for the difference in heat transfer. Figure 8 illustrates the schematic diagram of the gas–liquid flow inside the two composite porous structures. The schematic diagram of the gas–liquid flow in a surface microporous composite porous structure is shown in Figure 8a. Bubbles grow along the heating surface and escape upward from the center of the large channel. The microporous layer on the surface of the sample promotes nucleation, and the liquid is supplied downward along the surface of the sample skeleton to the heating surface. The surface microporous composite porous structure increases the heat transfer area and nucleation sites at low heat flux, resulting in a higher heat transfer coefficient. In the high heat flux region, the millimeter channel should not only bear the effective supply of liquid but also bear the escape of bubbles, which limits its heat transfer enhancement under high heat flux. Robinson et al. [31] found that the effective pore size of several tens of micrometers inside the porous skeleton sample can achieve better capillary performance and permeability. The pores inside the SF-1 sample discussed in this paper demonstrate favorable interconnectivity, thereby facilitating a harmonious interplay between the capillary performance and the permeability. For the framework microporous composite porous structure, the micropores within the framework act as supplementary pathways for liquids, while macroscopic pores become the main pathway for bubble escape (Figure 8b). In the high heat flux region, the micropores in the framework microporous composite porous structure form different divisions of labor from macroscopic pores. Consequently, compared to the surface micropore composite porous structure, the framework micropore composite porous structure can achieve a higher heat transfer coefficient in high heat flux regions.

**Figure 9:**
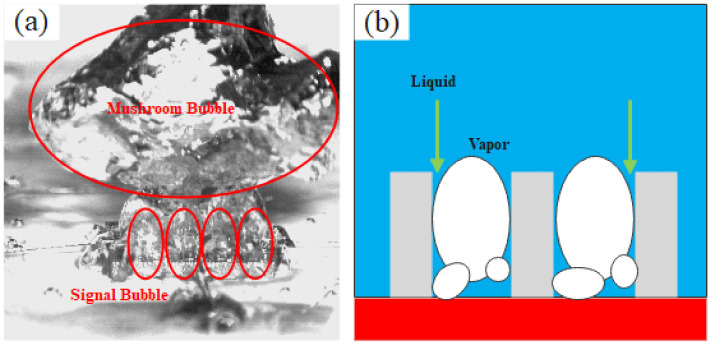
Bubble motion in composite porous structure. (**a**) High-speed photos of bubble motion. (**b**) Schematic diagram of gas–liquid motion.

As previously observed, augmenting the microporosity on the surface or inside the framework of the 3D-printed sample through a controlled printing process parameters enhances the HTC of the composite porous sample but has no significant impact on the CHF improvement. Due to the presence of large pores within the sample, bubbles are confined within square cavities, impeding their coalescence and departure from the heating surface at a higher frequency compared to the smooth surface. This subsequently enhances the effective replenishment of the liquid, thereby improving the heat transfer efficiency of the heating surface. As the heat flux increases, the size of the initial bubbles generated on the heating surface also correspondingly increases [32]. The large square pores in the composite porous structures can effectively constrain bubble coalescence, which is the key to improving the CHF of composite porous structures. Figure 9 depicts a photo of the high-speed motion of bubbles in a composite porous structure and a schematic diagram of gas–liquid flow.

In this study, the bubble departure diameter was calculated by Fritz’s equation [33].

(7) $$D_{b}=0.0208\theta {\left[\frac{\sigma }{g(\rho_{l}-\rho_{v})}\right]}^{1/2}$$

where *θ* is the contact angle, *σ* is the surface tension force, *g* is the gravitational force, and *ρ_l_* and *ρ_v_* are the density of the liquid and the vapor, respectively.

It is postulated that at saturated atmospheric conditions, the departure diameter of the water boiling bubbles on the heating surface calculated by Equation (7) should be approximately equal to the length of the groove to prevent the lateral coalescence of bubbles. The calculation of the bubble departure diameter is calculated at 2.02 mm by using water physical parameters at 100 °C and contact angle (*θ* = 38.8°). The dimension of the square pore is larger than that of a single square pore, which can effectively limit the escape of bubbles. The macroscopic pore size of the current composite porous structure is consistent, leading to consistent confinement effects on the escaping bubbles. Micropores on the surface or inside the framework of composite porous structures are crucial factors for HTC enhancement, while the large-sized pore structure is a key factor limiting CHF.

## 4. Conclusions

Composite porous structures with different pore forms were fabricated by selective laser melting technology, and the influence of pore forms on the boiling heat transfer characteristics of composite porous structures was investigated through pool boiling experiments. The main conclusions are as follows:

(1) Surface micropores play a crucial role in affecting the HTC of composite porous samples, and increasing the surface roughness of the sample can enhance the HTC. Sandblasting treatment removes the microporous layer on the surface of the composite porous sample, resulting in a decrease in the HTC of the sample. In the present study, the average heat transfer coefficient of the sample with a rough surface was increased by 40% compared with that of the sandblasted sample.
(2) Under low heat flux conditions, the surface micropores of composite porous samples increase the heat transfer area and nucleation sites, leading to a higher enhancement of HTC. Conversely, under high heat flux, composite porous samples with micropores in the framework can form effective gas–liquid separation, resulting in higher HTC enhancement.
(3) Increasing either the surface micropores or the framework micropores in the sample contributes to the HTC enhancement of the composite porous sample, and the limitation of macroscopic pores on bubbles is a key control factor for CHF enhancement.

## Figures and Tables

**Table 1:** Printing process parameters of different samples.

Test Number	SS-1	SS-2	SS-3	SF-1
Power (W)	180	180	180	180
Speed (mm/s)	1000	1000	1000	1000
Layer thickness (mm)	0.02	0.02	0.04	0.02
Distance (mm)	0.08	0.08	0.08	0.2

## Data Availability

Not applicable.

## Nomenclature

*A*	surface area, mm^2^
*D_b_*	bubble diameter, mm
*g*	gravitational acceleration, m/s^2^
*h*	heat transfer coefficient, W/(cm^2^·K)
*I*	current, A
$q^{\prime \prime }$	heat flux, W/cm^2^
*T*_*sat*_	saturated temperature of the working fluid, K
*U*	voltage, V
Greek symbols	
σ	surface tension force, N/m
ρ	density, kg/m^3^
θ	contact angle, °
Subscripts	
*b*	bubble
*l*	liquid
*sat*	saturation
v	vapor
w	wall
